# Pneumococcal VncR Strain-Specifically Regulates Capsule Polysaccharide Synthesis

**DOI:** 10.3389/fmicb.2019.02279

**Published:** 2019-10-01

**Authors:** Prachetash Ghosh, Masaud Shah, Subramaniyam Ravichandran, Sang-Sang Park, Hamid Iqbal, Sangdun Choi, Kyeong Kyu Kim, Dong Kwon Rhee

**Affiliations:** ^1^School of Pharmacy, Sungkyunkwan University, Suwon, South Korea; ^2^Department of Molecular Science and Technology, Ajou University, Suwon, South Korea; ^3^Department of Molecular Cell Biology, School of Medicine, Sungkyunkwan University, Suwon, South Korea

**Keywords:** VncR, capsular polysaccharide, strain-specific, lactoferrin, *Streptococcus pneumoniae*

## Abstract

Capsular polysaccharides (CPS), a major virulence factor in *Streptococcus pneumoniae*, become thicker during blood invasion while not during asymptomatic nasopharyngeal colonization. However, the underlying mechanism controlling this differential pneumococcal CPS regulation remain unclear. Here, we show how VncR, the response regulator of the vancomycin resistance locus (*vncRS* operon), regulates CPS expression in *vncR* mutants in three serotype (type 2, 3, and 6B) backgrounds upon exposure to serum lactoferrin (LF). Comparative analysis of CPS levels in the wild type (WT) of three strains and their isogenic *vncR* mutants after LF exposure revealed a strain-specific alteration in CPS production. Consistently, VncR-mediated strain-specific CPS production is correlated with pneumococcal virulence, *in vivo*. Electrophoretic mobility-shift assay and co-immunoprecipitation revealed an interaction between VncR and the *cps* promoter (*cpsp*) in the presence of serum. In addition, *in silico* analysis uncovered this protein-DNA interaction, suggesting that VncR binds with the *cpsp*, and recognizes the strain-specific significance of the tandem repeats in *cpsp*. Taken together, the interaction of VncR and *cpsp* after serum exposure plays an essential role in regulating differential strain-specific CPS production, which subsequently determines strain-specific systemic virulence. This study highlights how host protein LF contributes to pneumococcal VncR-mediated CPS production. As CPS plays a significant role in immune evasion, these findings suggest that drugs designed to interrupt the VncR-mediated CPS production could help to combat pneumococcal infections.

## Introduction

*Streptococcus pneumoniae*, usually a commensal in the upper human respiratory tract, is an etiologic agent of pneumonia, sepsis, and meningitis (Bogaert et al., 2004). The successful alteration of a harmless commensal to an invasive pathogen is associated with the trafficking of bacteria across tissue barriers and the subsequent bacterial adaptation to altered host niches. This progression is multifunctional and demands strict regulation (Hammerschmidt et al., 2005).

Among the pneumococcal virulence factors, the capsular polysaccharide (capsule, CPS) is the most significant, and is responsible for disease pathogenesis (Kadioglu et al., 2008). CPS shows a differential expression profile between carriage pneumococci and planktonic pneumococci, capable of causing an invasive disease (Wu et al., 2016). Although several proteins, such as CpsR (Wu et al., 2016), and ComE (Zheng et al., 2017) have been shown to play key roles in the regulation of *cps* genes, the underlying mechanism is poorly delineated. To date, 94 pneumococcal CPS have been reported (Nurse-Lucas et al., 2016), all of these except two are produced by a Wzy-polymerase-dependent mechanism (Tuomanen et al., 2004; Nurse-Lucas et al., 2016; Zheng et al., 2017). In contrast, the synthesis of the other CPS types (3 and 37) is mediated by a single membrane-bound glycosyltransferase. In these pneumococcal serotypes, the conserved sequences positioned at the 5′ end of all the other loci, which are responsible for the transcription of regulatory proteins, are either absent (type 37) or mutated (type 3) (Moscoso and García, 2009). The *cps* loci of all Wzy serotypes are positioned at the same chromosomal region (Zheng et al., 2017). *cps* locus promoter sequences (*cpsp*) and the first four genes *cpsA* to *cpsD* are highly conserved and take part in CPS regulation, whereas, genes downstream of *cpsD* are serotype-specific (Wu et al., 2016; Ghosh et al., 2018). Moreover, previous studies confirmed that the *cps* genes are transcribed as an operon from a sole promoter (Guidolin et al., 1994; Aanensen et al., 2007). In contrast, type 3 pneumococcal *cpsp* is completely different from the other serotypes (Caimano et al., 1998), as a short 87 bp region embracing the *cpsp* is strictly conserved only among the Wzy serotypes (Moscoso and García, 2009).

The two component signal transduction systems (TCSs) in bacteria are comprised of a membrane-bound histidine kinase protein (HK) and a cytosolic response regulator (RR) (McCluskey et al., 2004). Activation of the TCS by various stimuli causes HK to undergo autophosphorylation, which subsequently transfers a phosphate group to the RR. The phosphorylated RR leads to adaptive responses by altering gene expression (Finlay and Falkow, 1997). TCS10, also known as VncRS, is induced in vancomycin-tolerant clinical pneumococcal samples (Sung et al., 2006), whereas, mutations in *vncR* did not alter the pneumococcal virulence (Throup et al., 2000), indicating that the role of VncRS in *S. pneumoniae* virulence is complex and must be explicated.

Previously, we showed that the VncS ligand, serum lactoferrin (LF), induced the type 2 pneumococcal *vncRS* operon and augmented *in vivo* mortality rates mediated by *pep27*, a gene belonging to *vncRS* operon (Lee et al., 2018). Further, the expression of the *cps2A* gene, representing the extent of pneumococcal *cps* transcription, was upregulated in the presence of serum (Ogunniyi et al., 2002). Moreover, BLAST searches revealed that the DNA binding domain (DBD) of VncR is almost homogenous, whereas the *cps* locus consists of a large number of type-specific genes (McCluskey et al., 2004; Zheng et al., 2017). These considerations have raised our interest in studying the role of VncR in strain-specific CPS-mediated systemic virulence.

Here, we show that VncR regulates CPS synthesis in a strain-specific manner in the presence of LF, which is further associated with pneumococcal virulence. According to our knowledge, we report, for the first time, using both *in vitro* and *in silico* analysis, that VncR binds to the *cpsp* strain-specifically and regulates its synthesis during serum exposure.

## Materials and Methods

### Bacterial Strains and Growth Conditions

All the reagents used for bacterial culture were purchased from Difco BD (NJ, United States). *S. pneumoniae* strains D39 (type 2; GenBank: CP000410.2), WU2 (type 3; GenBank: U15171.1), and BG7322 (type 6B; GenBank: JF911505.1) were grown in THY medium (Todd Hewitt medium with 0.5% Yeast extract) at 37°C without aeration. *S. pneumoniae* strains possessing the *erm* marker were grown in media supplemented with 2.5 μg/ml erythromycin. In order to see the effect of the human serum or LF on CPS production, the strains were allowed to grow in THY broth until logarithmic phase (OD_550_ of 0.30) when 10% human serum or 30 mM LF was added and then co-incubated for certain periods. All the strains used in this study are listed in Table 1.

**TABLE 1:** Strains and plasmids used in this study.

**Strains/Plasmids**	**Characteristics**	**References**
**Strains (*S. Pneumoniae*)**		
D39	Type 2, encapsulated	Avery et al., 1944
WU2	Type 3, encapsulated	Dillard et al., 1995
BG7322	Type 6B, encapsulated	Elberse et al., 2011
D39Δ*vncR*	D39, an *ermB* cassette was inserted into the *vncR*	Lee et al., 2018
WU2Δ*vncR*	WU2, an *ermB* cassette was inserted into the *vncR*	This study
BG7322Δ*vncR*	BG7322, an *ermB* cassette was inserted into the *vncR*	This study
**Strains (*E. Coli*)**		
XL1-Blue	recA1 endA1 gyrA96 thi-1 hsdR17 supE44 relA1 lac [F’ proAB lacIqZΔM15 Tn10 (Tetr)]	Stratagene
BL21 (DE3)	gal (cI ts857 ind1 Sam7 nin5 lacUV5-T7 gene 1)	Novagen
**Plasmids**		
pHis-parallel 2	5517 bp; *E. coli* plasmid	Sheffield et al., 1999

### Construction of *vncR* Deletion Mutants

The *S. pneumoniae* D39 *vncR* deletion mutant was constructed in our previous study (Lee et al., 2018). Similarly, in this study, in WU2 and BG7322 pneumococcal strains, a donor DNA fragment in which the deleted *vncR* genes were substituted with an erythromycin resistance cassette (*ermB*), was created by overlapping PCR and incorporated into pneumococcal chromosomes by homologous recombination (Kim et al., 2012). The primers used here were listed in Supplementary Table S1.

### qRT-PCR

For a comparative study of the transcript levels of all genes under the *cps* locus of wild type (WT) strains and their respective *vncR* deletion mutants after serum or LF supplementation, the strains were subjected to RNA isolation using the Trizol method (Invitrogen, United States). cDNA was prepared using 1 μg of total RNA following the manufacturer’s protocol (Takara Clontech, CA, United States). Real time PCR, consisting of 50 ng of cDNA, 10 pmol of each primer (Supplementary Table S2), and SYBR Green master mix (Elpis, Seoul, Korea), was carried out in a 20 μl final volume with the StepOnePlus Real-Time PCR System (Applied Biosystems, CA, United States) under the following cycling conditions: 95°C for 10 min followed by 40 cycles at 95°C for 15 s, 55°C for 30 s, and 72°C for 30 s. All qRT-PCR data were normalized to the mRNA level of 16s rRNA genes used as endogenous controls.

### Analysis of Pneumococcal CPS by Enzyme-Linked Immunosorbent Assay (ELISA)

Indirect capsule ELISA with slight modifications were performed, as described previously (Xayarath and Yother, 2007), to determine the change in total CPS quantity in pneumococcus strains after serum treatment. *S. pneumoniae* strains were grown in THY up to A_550_ = 0.3, and treated with 10% human serum or LF for the desired period. The bacterial cells were pelleted down by centrifugation at 10,000 × *g* for 10 min, and washed thrice with sterile PBS. All bacterial samples were then normalized to the same optical density at 550 nm. After that, all the samples were heat-killed at 60°C for 20 min. These samples were coated into wells of microtiter plates (Corning Inc.) and incubated for overnight at 4°C. The wells were further blocked for 1 h with 1% (w/v) bovine serum albumin (BSA; Sigma-Aldrich) in PBS (BSA-PBS). After washing thrice with 0.5% Tween-20- containing PBS (PBST), 100 μl serotype-specific CPS rabbit antiserum (Statens Serum Institut, Copenhagen, Denmark) diluted 1:1,000 with BSA-PBS and incubated at room temperature for 1 h. After washing, these samples were incubated with a 1:5,000 dilution of the horseradish peroxidase (HRP) conjugated secondary rabbit antibody at room temperature for 1 h, followed by washing with PBST. Then, the TMB substrate was added to the wells, and the A_450_ was determined by an ELISA reader (Softmax).

### Fluorescence Microscopy

CPS was further observed using fluorescence microscopy as described previously, with slight modifications (Echlin et al., 2016). Different *S. pneumoniae* strains were grown in THY broth, and after serum treatment, bacteria were collected during the logarithmic phase of growth by centrifugation. Subsequently, the cells were fixed by 1 h incubation with 4% paraformaldehyde, then incubated with 1:100 dilution of the serotype-specific rabbit antiserum (Statens Serum Institut, Copenhagen, Denmark) in 5% FBS and 0.2% saponin for 1 h at room temperature, and then reacted with a 1:200 dilution of Alexa 488 secondary antibody (Thermo Fisher Scientific, MA, United States) for 1 h at room temperature. The sample (15 μl) was mounted on a glass slide and viewed using a confocal microscope (Carl Zeiss LSM 510, Oberkochen, Germany).

### Survival Study

Four weeks old male CD1 mice were purchased from Koatech (Pyeongtaek, Korea). Experiments were carried out in a manner which reduces animal suffering and we also used the least numbers associated with effective statistical evaluation. All experimental procedures were sanctioned by the Ethical Committee of Sungkyunkwan University, based on the Helsinki Declaration. To evaluate the role of VncR-regulated CPS thickness on virulence, mice were intranasally infected (*n* = 10) or intraperitoneally infected (*n* = 15) with 5 × 10^7^ and 1 × 10^5^ cells, respectively, of WT and their respective *vncR* deletion mutants after anesthesia with ketamine-xylazine mixture. Mice were observed each day at 4 h intervals, and the survival time for each mouse was recorded.

### Protein Purification

VncR comprises of two distinct functional domains, designated N-terminal receiver and C-terminal effector domains. Here, the VncR C-terminal DNA binding domain (VncRc) was purified. The VncRc-coding portion of the gene (position 119–242) was amplified using polymerase chain reaction (PCR) from the D39 WT genomic DNA. The PCR-amplified DNA was restriction enzyme-digested using *Nco*I/*Xho*I, cloned into the *Nco*I/*Xho*I sites of the pHis Parallel 2 vector and transfected into *Escherichia coli* XL1-blue and then, into *E. coli* BL21 (DE3) for overexpression. Recombinant strains were grown at 37°C to an optical density (OD)_600_ of 0.6–0.8 in Luria Bertani (LB) medium, supplemented with 50 μg/ml ampicillin, with 150 rpm shaking. Protein expression was induced by supplementing the culture medium with 0.5 mM β-D-1-thiogalactopyranoside (IPTG) at 25°C for 24 h. Following this, cells were collected by centrifugation at 3,382 × *g* for 15 min at 4°C, resuspended in default buffer [50 mM Tris-HCl at pH 7.5, 150 mM NaCl, 5 mM β-mercaptoethanol, and 1 mM phenylmethylsulfonyl fluoride (PMSF)]. The cells were lysed by sonication, and centrifugation at 16,000 × *g* for 45 min was performed to separate cellular debris and insoluble proteins. Ni-NTA resin (GE Healthcare, United Kingdom) was used to purify supernatant protein fractions as previously described (Sheffield et al., 1999). His-tagged VncRc was then eluted using elution buffer (50 mM Tris-HCl at pH 7.5, 500 mM NaCl, 200 mM Imidazole). The protein was further concentrated by using a centrifugal filter (Centricon, Millipore, Germany) to 5 mg/ml, and then the quality of the purified protein was confirmed by SDS-PAGE.

### Electrophoretic Mobility-Shift Assay (EMSA)

DNA fragments of the *cpsp* of three independent pneumococcal strains were generated by PCR using the respective genomic DNA of those strains as templates (Primers listed in Supplementary Table S3). DNA (200 ng) was incubated with equal amount of purified VncRc protein in buffer containing 10 mM Tris HCl pH 7.9, 10 mM MgCl_2_, 50 mM NaCl, 100 μg/ml bovine serum albumin (BSA), and 10% serum (Sigma-Aldrich., St. Louis, MO, United States). After a 5 min incubation at 37°C, the samples were loaded on a 6% native polyacrylamide (PAGE gel) pre-run and electrophoresed at 80 and 100 V, respectively, for 30 min at room temperature. The gels were washed once with water and stained using SYBR Gold (Thermo Fisher Scientific, MA, United States).

### Western Blotting

The bands from EMSA gel were excised out and subjected to “TCA (trichloroacetic acid) protein precipitation method” (Koontz, 2014). The TCA-precipitated proteins were mixed with 2X SDS-PAGE loading buffer and loaded on a 15% SDS-PAGE gel run at 200 V for 45 min. The gel was subjected to Western Blot transfer in Towbin buffer at 200 V for 1 h. After the transfer was completed, the membrane was blocked with 5% skim milk powder in TBST (Tris-buffered saline with Tween-20) for 1 h followed by overnight incubation with anti-VncR primary antibody “(Kindly provided by Professor Elaine I. Tuomanen)” in 1:2000 dilution. After washing thrice for 10 min with TBST, the membrane was incubated with an anti-rabbit secondary antibody at 1:5000 dilution followed by washing thrice for 10 min with TBST and developed using the picoEPD western blot detection kit (ELPIS-Biotech, Korea).

### Co-immunoprecipitation (Co-IP)

For Co-IP experiments, 200 ng of DNA was incubated at 37°C with an equal amount of His-tagged VncRc protein in buffer, containing 10 mM Tris HCl pH 7.9, 10 mM MgCl_2_, 50 mM NaCl, 100 μg/ml BSA, and 10% serum (Sigma-Aldrich, St. Louis, MO, United States). Anti-His antibody was added after 5 min incubation, and the mixture was incubated overnight at 4°C, with gentle agitation. The next day, protein-A agarose beads were added to the mixture and incubated further for 4 h at 4°C under shaking. The mixture was centrifuged at 370 × *g* for 2 min and the supernatant was discarded. The pellet was washed with 100 μl H_2_O and the supernatant was discarded. The pellets were resuspended in the remaining buffer and 10 μl of the Co-IP mix was used for PCR to identify the presence of the *cpsp* using specific primers. PCR was performed for 35 cycles, with an annealing temperature of 60°C, and the products were run on a 1% agarose gel, stained with ethidium bromide, and visualized using a GelDoc system (Bio-Rad, CA, United States).

### Modeling and Protein-DNA Interface Analysis

To predict the 3D structure of the VncR full-length protein, we utilized three different tools; MOE (2018), I-TASSER and SwissModel. These packages predicted almost similarly-folded structures of the VncR with RMSD ranging from 0.5 to 1 Å. These models were further evaluated and optimized to remove all kinds of abnormalities. To predict the DNA binding mechanism of VncR, the fully optimized structure modeled by I-TASSER server was used in protein-DNA docking. The 3D structure of the VncR-binding region of *cpsp*, containing direct repeats, was modeled through 3D-DART and MOE, and further optimized (using default parameters in MOE). To evaluate the possible binding mechanism of the DBD of VncR and *cpsp*, the receiver/regulatory domain of the VncR was removed and subjected to protein-DNA docking using ZDOCK online server (Chen et al., 2003) and MOE. Based on the electrostatic complementarity, geometry, and hydrophobicity of the molecular surface, ZDOCK ranks the 100 most probable predictions out of thousands of candidates and has been considered among the top docking servers.

### Molecular Dynamics Simulations

To estimate structural changes in the DNA-VncR DBD as a function of time, molecular dynamics simulations (MDS) has been widely utilized. The most accurate and validated complex was simulated in GROMACS v5.0.7 (Abraham et al., 2015). The system was solvated in a dodecahedron box using TIP3P waster model (Jorgensen et al., 1983). The AMBER99SB-ILDN force field has been improved for protein-DNA complexes (Lindorff-Larsen et al., 2010). To mimic the infinite system, periodic boundary conditions were applied to the box in all directions. For long-range electrostatics, the Particle Mesh Ewald approach was employed using 10Å cutoff values (Darden et al., 1993). Bond lengths were constrained using LINC algorithm (Hess et al., 1997), and the system was neutralized using counter ions. Additionally, 100 mM NaCl concentrations were added to mimic physiological conditions. Steepest descents and/or conjugate gradient minimization with a maximum tolerance of 100 kJ/mol/nm were performed to remove steric interactions. To equilibrate the system, it was initially simulated for 100 ps under a constant volume (NVT) ensemble to achieve 300 K by the V-rescale method (Bussi et al., 2007). The equilibrated structures from the NVT ensemble were subjected to constant pressure (NPT) equilibration for 100 ps using the Parrinello-Rahman barostat under an isotropic pressure of 1.0 bar (Parrinello and Rahman, 1981). During data collection, the V-rescale thermostat and Parrinello-Rahman barostat were used to maintain the temperature and pressure at 300 K and 1 bar, respectively, and the atomic coordinates were saved every 2.0 ps for 100 ns total simulation time.

### Data Analysis and Graphics

Most of the data were analyzed with built-in modules of GROMACS v5.0.7. Graphical images were produced with PyMol (Rigsby and Parker, 2016) and VMD (Humphrey et al., 1996). Interface analyses were performed in MOE, VMD, and UCSF Chimera, and images were generated in MOE and PyMol. For alanine scanning mutagenesis, a similar protocol was used as described elsewhere (Shah et al., 2016). All computational studies were performed on a Dell PowerEdge server with a CentOS6 GNU/Linux operating system.

### Statistical Analysis

Differences in overall survival rates between groups were analyzed by the Log-rank test. Each experiment was carried out at least thrice in duplicates and results were expressed as mean ± standard error of the mean (SEM). Statistical analysis was performed by ANOVA using the Graph Pad Prism software (version 5, Graph Pad Software Inc, CA, United States) and *P* < 0.05 were considered to be statistically significant.

## Results

### Strain-Specific Induction of *vncR* by Serum

**FIGURE 1:**
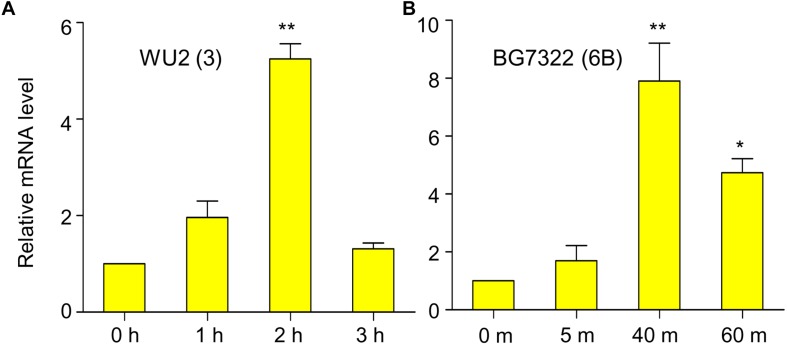
Serum induces vncR expression in a serotype-specific manner. *S. pneumoniae* strains WU2 **(A)** and BG7322 **(B)** were cultured in the THY broth up to mid-log phase, and then incubated with 10% humanprotect serum for 3 h and 60 min respectively. The mRNA expression levels of *vncR* were analyzed by qRT-PCR. Data are expressed as mean ± standard error of mean (SEM) of 3 experiments in quadruplicates. *^∗^P* < 0.05, *^∗∗^P* < 0.01 (One-way ANOVA) as between compared groups.

In our previous study, mRNA levels of D39 (type 2) *vncR* were maximally induced by 5 min treatment of 10% human serum (Lee et al., 2018), however, 5 min exposure to 10% serum failed to activate *vncR* mRNA levels in BG7322 (type 6B) and WU2 (type 3) pneumococci (data not shown). Nonetheless, subsequent prolonged treatment with 10% serum revealed maximal induction of the *vncR* mRNA in types 3 and 6B in 2 h and 40 min, respectively (Figure 1).

### VncR Mediates *cps* Gene Transcription in a Strain-Specific Manner During Serum Exposure

**FIGURE 2:**
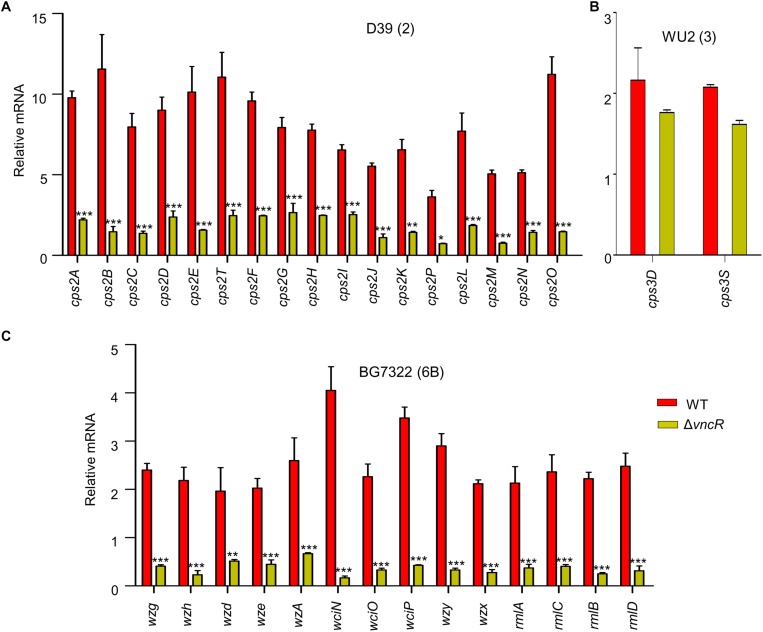
VncR regulates *cps* gene transcription in a strain-specific manner after serum exposure. *S. pneumoniae* strains D39 (type 2; **A**), WU2 (type 3; **B**), and BG7322 (type 6B; **C**) and their respective *vncR* deletion mutants were cultured in THY broth up to mid-log phase, and then incubated with 10% human serum for 5 min, 2 h, and 40 min respectively. The mRNA expression levels of the *cps* operon were analyzed by qRT-PCR. Data are expressed as mean ± standard error of mean (SEM) of 3 experiments in quadruplicates. *^∗^P* < 0.05, *^∗∗^P* < 0.01, *^∗∗∗^P* < 0.001 (Two-way ANOVA) as between compared groups.

To assess whether the expression of the *cps* genes is transcriptionally regulated by VncR after serum exposure, comparative qRT-PCR was carried out for all genes under the *cps* loci, D39 (17 genes), WU2 (2 genes), and BG7322 (14 genes), and their respective isogenic *vncR* deletion mutants. A schematic representation of these three types of *cps* loci has been given in Supplementary Figure S1. Results showed that in D39, all the 17 genes were transcriptionally induced ∼4–12-folds within 5 min of serum exposure, which was significantly higher than the induction of its isogenic Δ*vncR cps* genes (Figure 2A).

In type 3, only two genes, *cps3D* and *cps3S*, were shown to be responsible for CPS synthesis (Caimano et al., 1998). We found that after 5 min of serum exposure, these *cps* genes became up-regulated about ∼1.6–2.1-folds from their respective basal levels in both the WT WU2 strain and its Δ*vncR* mutant (data not shown). Comparative transcription levels of their *cps* genes were studied for up to 2 h of serum exposure, but they did not show any significant differences between WU2 WT and WU2Δ*vncR* (Figure 2B).

Among the 14 genes in the *cps* locus involved in CPS synthesis in type 6B (Elberse et al., 2011), there were no significant differences between BG7322 and its Δ*vncR* mutant after 5 min of serum exposure (data not shown). After 40 min exposure to the serum, *cps* genes expression in the WT were upregulated, whereas that of its isogenic Δ*vncR* showed significant reduction (Figure 2C). These results suggest that pneumococcal *cps* genes could be regulated strain-specifically by VncR.

### VncR Regulates CPS Production in *S. pneumoniae* Strain-Specifically

**FIGURE 3:**
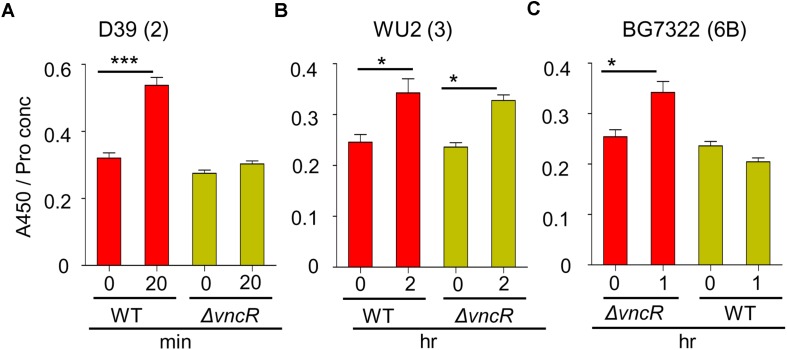
VncR regulates pneumococcal CPS levels in a strain-specific manner after serum exposure. Pneumococcal strains WT D39 **(A)**, WU2 **(B)**, and BG7322 **(C)** and their isogenic *vncR* deletion mutants were treated with serum for 20 min, 2 h, and 1 h, respectively. Total CPS quantities in whole cell lysates were determined by ELISA using serotype-specific antiserum. All samples were assayed in quadruplicates and each assay was repeated thrice. Data are expressed as mean ± standard error of mean (SEM) of 3 experiments in quadruplicates. *^∗^P* < 0.05, *^∗∗∗^P* < 0.001 (One-way ANOVA) as between compared groups.

To confirm the effect of VncR on type 2, 3, and 6B CPS production, CPS quantities were estimated by indirect ELISA, and visualized by confocal microscopy. ELISA results showed that total CPS amounts were significantly increased after 20 min of serum exposure in D39, whereas, no significant changes were observed in D39Δ*vncR* (Figure 3A). Unlike in the D39 strain, even after 2 h of serum treatment, CPS amounts in the WU2 WT and its Δ*vncR* mutant did not show any significant differences, indicating that *vncR* deletion in type 3 does not affect CPS production (Figure 3B), further supporting our qRT-PCR data.

As our qRT-PCR analysis showed maximal differences in the *cps* mRNA levels of BG7322 WT and Δ*vncR* mutant after 40 min of serum exposure, the total CPS amounts were compared between these two strains, for up to 1 h of serum exposure, using ELISA. Interestingly, the CPS level in the BG7322 WT was significantly increased, whereas that in the Δ*vncR* did not change significantly (Figure 3C).

To reconfirm strain-specific CPS regulation by the VncR, the WT D39, WU2, and BG7322, and their respective Δ*vncR* mutants were grown to log phase and then treated with 10% human serum for a specified time for evaluation using confocal microscopy. Microscopy data revealed that D39 showed stronger fluorescence after 20 min serum exposure, whereas its Δ*vncR* mutant did not (Supplementary Figure S2). In contrast, no significant differences in fluorescence levels were found between the WU2 and BG7322 WTs, and their respective Δ*vncR* mutants (data not shown).

### LF Is the Differential VncR Regulator for CPS Transcription and Production

**FIGURE 4:**
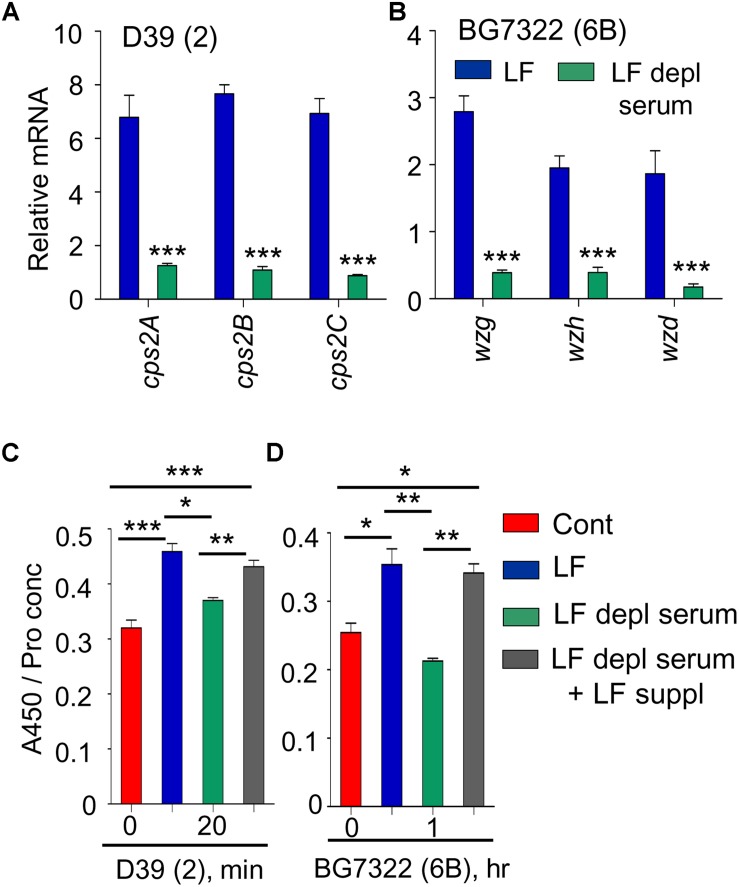
VncR regulates *cps* gene transcription and CPS levels in a strain-specific manner after serum LF exposure. The D39 **(A)** and BG7322 **(B)** WT strains were incubated for 5 and 40 min, respectively, with LF and in LF-depleted serum. The mRNA expression levels of the *cps* genes were analyzed by qRT-PCR. Additionally, the WT D39 **(C)** and BG7322 **(D)** strains were incubated for 20 min and 1 h, respectively, with LF and in the LF-depleted serum and LF was also exogenously added to the LF-depleted serum. Total CPS quantities in whole cell lysates were determined by ELISA using serotype-specific antiserum. All samples were assayed in quadruplicates and each assay was repeated thrice. Data are expressed as mean ± standard error of mean (SEM) of 3 experiments in quadruplicates. *^∗^P* < 0.05, *^∗∗^P* < 0.01, *^∗∗∗^P* < 0.001 (One-way ANOVA) as between compared groups.

Previously, supplementation of 30 mM LF caused the induction of type 2 pneumococcal *vncRS*, proposing LF as a VncS sensor ligand (Lee et al., 2018). Thus, the effect of LF supplementation was examined in D39, WU2, and BG7322 strains. *cps* genes in D39 and BG7322 (only representative genes shown here) were induced significantly after 5 and 40 min of 30 mM LF exposure, respectively, whereas, they were not induced by LF depleted serum treatment (Figures 4A,B). ELISA results consistently indicated significant augmentation of total CPS levels in the D39 and BG7322 WT, after 20 min and 1 h of LF serum supplementation, respectively, whereas, no substantial changes were observed after exposure to LF-depleted serum (Figures 4C,D). Moreover, the addition of exogenous LF to the LF-depleted serum resulted in a significant increase in CPS levels (Figures 4C,D), establishing the role of LF in VncR-mediated CPS production during serum exposure. As expected, LF treatment did not show any effect on the CPS production of WU2 strain (Supplementary Figure S3).

### VncR Regulates Pneumococcal Virulence *in vivo* in a Strain-Specific Manner

**FIGURE 5:**
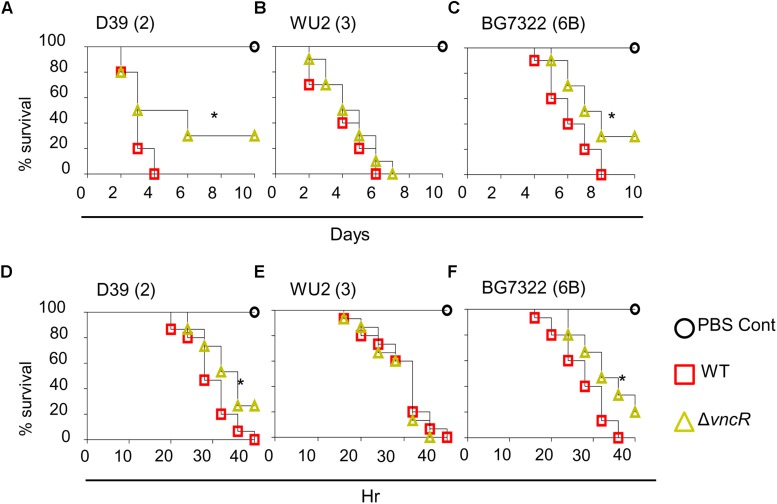
VncR modulates *in vivo* virulence in a strain-specific manner. Mice (*n* = 10) were infected intranasally (*i.n*) with ∼5 × 10^7^ CFU of D39 **(A)**, WU2 **(B)**, BG7322 **(C)** or isogenic mutants, and survival times were determined. Mice (*n* = 15) were infected intraperitoneally (*i.p*) with 1 × 10^5^ CFU of D39 **(D)**, WU2 **(E)** BG7322 **(F)** or their isogenic *vncR* mutants. Mice were observed for 40 h and survival was assayed every 4 h. The results are representative of at least three independent experiments. *^∗^P* < 0.05 (Log-rank test) as between compared groups.

To study the effect of Δ*vncR* on virulence, mice were infected with pneumococci intranasally (*i.n.*) and survival times were monitored. Survival rate of mice infected with D39Δ*vncR* was 30%, whereas, all D39WT-infected mice died within 4 days post-infection (Figure 5A). However, the survival rates of WU2Δ*vncR* infected group and WU2 WT showed no significant differences; all mice in both groups succumbed 7 and 6 days, post-infection, respectively (Figure 5B). Similar to type 2, the survival rate of the BG7322Δ*vncR*-infected group was 30% compared to 0% for the BG7322 WT-infected group (Figure 5C). For further confirmation, the effect of Δ*vncR* on virulence in a sepsis model was examined by intraperitoneal (*i.p*.) infection with D39, WU2, and BG7322 WTs and their Δ*vncR* mutants. Mice infected with the D39Δ*vncR* and BG7322Δ*vncR* mutants consistently showed significantly higher survival rates than mice infected with their respective WTs, 40 h post-infection (Figures 5D,F); whereas, mice infected with the WU2Δ*vncR* mutant showed no differences in the survival rates compared with the WU2 infected mice (Figure 5E). Overall, D39 and BG7322, comprising the Δ*vncR*, showed significantly reduced virulence than their respective WTs, while WU2Δ*vncR* seemed to be almost as equally virulent as its WT.

### VncR Binds to the *cpsp* in a Serum-Dependent Manner

**FIGURE 6:**
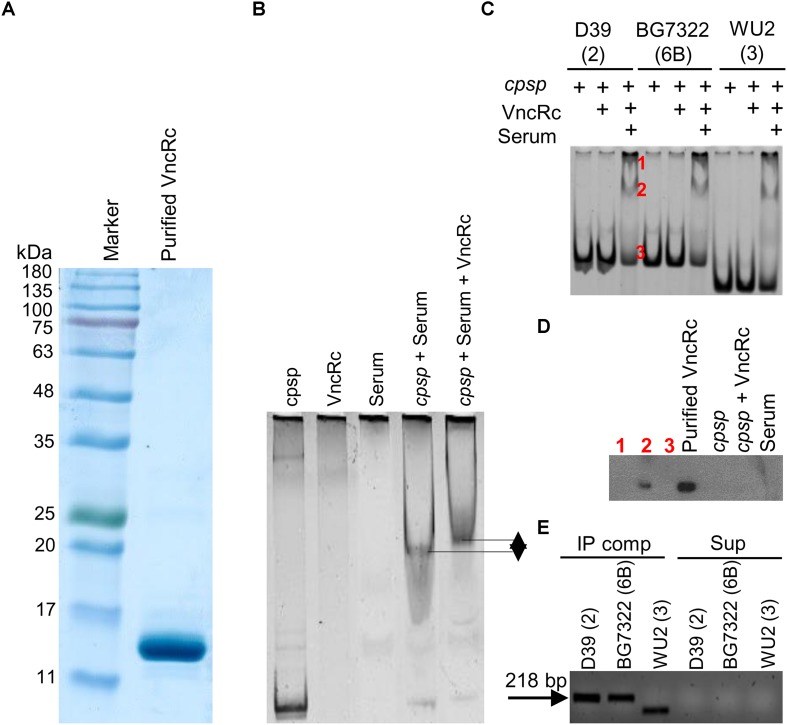
Characterization of the interaction between VncRc and the pneumococcal *cpsp*. SDS-PAGE analysis to check the purity of the His-tagged VncRc protein **(A)**. EMSA of 6 × His-VncRc protein and D39 *cpsp*, in the presence of serum. Control experiments have been done using only *cpsp* and serum. When VncRc was incubated along with *cpsp* and serum, the shift was more uplifted than the shift of *cpsp* and serum mix **(B)**. EMSA of 6 × His-VncRc protein and D39, BG7322, and WU2 *cpsp*, in the presence of serum. The 218 bp D39 and BG7322 *cpsp*, and 142 bp WU2 *cpsp* were independently incubated with purified VncRc. Results showed that all three types of *cpsp* can interact with VncRc in the presence of serum. Bands marked in red color as “1” and “2” are the resultant shifted bands, and band marked in red color as“3” is unshifted band after interaction of D39 *cpsp* and VncRc in presence of serum **(C)**. Western blot results using anti-VncR antibody of the bands marked in red color as “1,” “2,” and “3” in **(B)**. Purified VncRc, a band of *cpsp* alone (without VncR), an unshifted band after treatment with VncRc, and serum have been also used as controls. Confirmation of the presence of VncRc in shifted EMSA bands (marked as “2”) – only the shifted bands of D39 were used **(D)**. Detection of *cpsp* by PCR from the co-immunoprecipitated VncRc-*cpsp* complex (in the presence of serum) after immunoprecipitation using an anti-His antibody **(E)**. The results in **(B**–**E)** are representatives of three independent experiments.

To confirm whether the DBD of the VncR protein can bind to the promoter region of *cps*, we performed EMSA with the purified C-terminal DNA binding domain of VncR (VncRc, Figure 6A) and promoter region of *cps* genes. Our results revealed that in presence of serum, VncRc could bind to the 218 bp *cpsp* of types 2 and 6B, as well as, to the 142 bp type 3 *cpsp*. Interestingly, there were no shift of bands when we incubated *cpsp* and VncRc without serum (Figure 6C). Control experiments have been done using only *cpsp* and serum. As among all the strains tested here, the CPS of type 2 39 showed maximal induction after serum treatment within the shortest period, we have included the representative figure of this control experiment using *cpsp* of type 2 D39 and VncRc protein (Figure 6B). Presence of DNA binding proteins in the human serum is a well-established fact (Sylvia et al., 1975) since a long time, so it is quite possible that when *cpsp* and serum were incubated, *cpsp* (DNA) could bind to any of these proteins and showed the shift in the gel. But, when VncRc was incubated along with *cpsp* and serum, the shift was more uplifted than for the mix of *cpsp* and serum only. This result confirms that VncRc can bind to *cpsp* in the presence of serum. Subsequent western blotting using an anti-VncR antibody with these bands (D39 only) of EMSA confirmed the presence of VncRc in the complex (Figure 6D). To corroborate EMSA results, we performed a co-IP experiment using all three *cpsp* independently and His-tagged VncRc protein. The VncRc-*cpsp* complexes were pulled down using anti-His antibody and subsequent PCR results indicated the presence of specific *cpsp* in those complexes (Figure 6E), which was reconfirmed by sequence analysis (Supplementary Figure S4). The supernatants, after pulling down the co-IP complexes, were used as negative controls in PCR reactions.

### Modeling and Protein-DNA Docking Analyses

**FIGURE 7:**
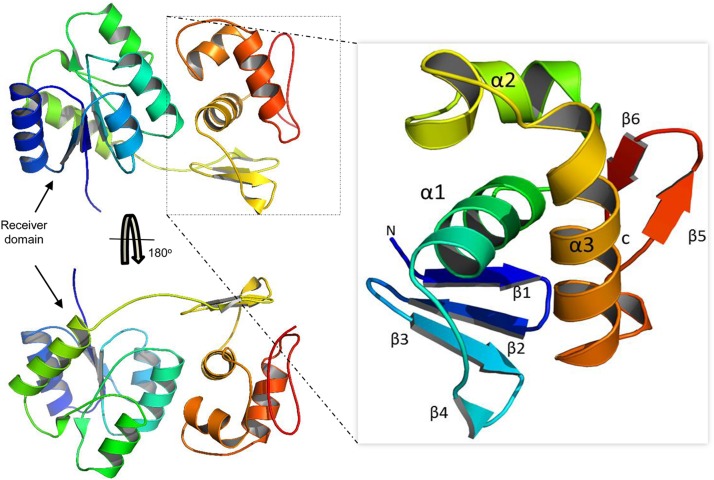
Domain organization of the VncR full-length protein. VncR contains an N-terminal receiver or regulatory domain and a C-terminal DNA-binding domain (DBD). The DBD has a typical helix-turn-helix fold exhibited by OmpR/PhoB subfamily proteins to recognize direct repeats in promoter regions.

Our model suggests that VncR shares sequence and structural similarity with response regulators, including PrrA, VraR, and KdpE, from different bacteria (Leonard et al., 2013; Narayanan et al., 2014). Overall, VncR acquired a stable, folded structure with two distinct domains; the N-terminal domain has a typical (β-α)_5_ folded structure (Figure 7) that has been reported as analogous to the other receiver domain-containing OmpR/PhoB subfamily proteins (Bourret, 2010). The C-terminal domain acquires a typical helix-turn-helix fold and contains four and two β-sheets at its N- and C-terminuses, respectively, sandwiching the DNA-recognizing helix-turn-helix structure (Figure 7 enhanced part).

**FIGURE 8:**
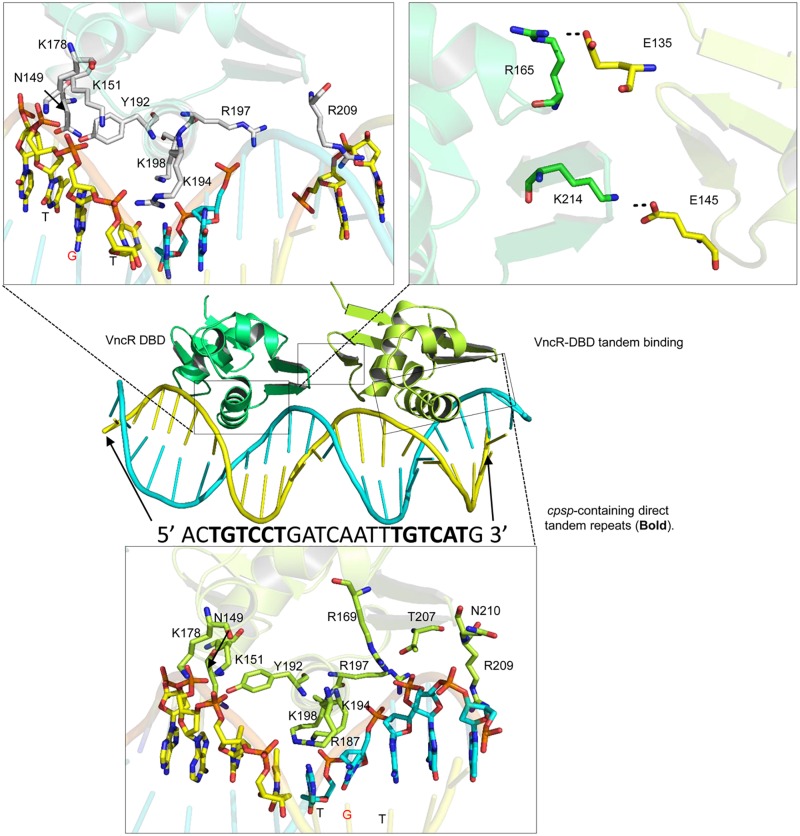
Tandem binding of VncR-DBD to the *cps*-direct repeat-containing promoter region. The α3 helix of VncR-DBD contains positively charged residues that recognize and interact with the direct repeat-containing *cps*-promoter in tandem. The salt bridges between the two DBD domains stabilize the tandem binding pattern.

Bacterial response regulators recognize the promoter DNA sequence and bind to the direct repeats in tandem (Blanco et al., 2002; Okajima et al., 2008). Similar direct repeats, containing TGTCC/AT, were found in the *cpsp* of types 2 and 6, but not in type 3 *S. pneumoniae*. Further, the second repeat in type 6 may contain adenosine (A) or thymine (T) instead of conserved guanosine (G) (Supplementary Figures S5A, S6). Docking procedures suggest that charged residues in the α3 helix established electrostatic interactions with the negatively charged backbone atoms of the major groves (Figure 8). Considering the “G” to “A/T” mutation in the second repeat of type 6, the “GC” base pair of type 2 *cpsp* was mutated into an “AT” base pair and analyzed for differences. Interestingly, we found that in the type 2 WT, the aromatic base of cytosine establishes a typical cationic-π interaction with a positively-charged amide group of Lys194. However, this interaction was lost in the mutant type 2 *cpsp* (Supplementary Figure S6). The methyl group moiety at the base of thymine creates a bulky hydrophobic patch (Supplementary Figure S6, electrostatic surface map) in the major grove of the *cpsp*, suggesting that, a “G” to “A/T” mutation could possibly reduce, or delay, VncR binding and thus CPS production.

### Molecular Dynamics Simulations (MDS) and Interface Analysis

The protein-DNA complex (using the type 2 template) was placed in physiological saline and subjected to 100 ns simulation. The RMSD plot suggests the overall stability of the complex with an average 3Å deviation (Supplementary Figure S5B). To track the structural dynamics and monitor any domain abnormality, the coordinates of the simulating system were extracted from the MD trajectory as a function of time and converted into animation (Supplementary Videos S1, S2). The lowest-energy representative complex was extracted from the MD trajectory and investigated for detailed interface analysis. The α3 helix, containing positively charged arginine (Arg187 and 197) and three lysine residues (Lys194, 198, and 199), established strong electrostatic interaction with the backbone phosphates as well as the basis. Besides, the inter-DBD interface is stabilized by salt bridges established between the Glu145 and Lys214, and Glu135 and Arg165 (Figure 8). A similar interaction pattern has been previously observed in structural studies of the response regulator, RstA (Li et al., 2014).

### Alanine Scanning Mutagenesis of the Interfacial Residues

Positively charged and other interfacial residues in the α3 helix were mutated into alanine (using the type 2 template) and their relative effect on the overall complex was measured in a tandem complex. Besides the positively charged residues in the α3 helix, Arg209 has a crucial role in DNA recognition and binding. Overall the DNA-binding affinity of the mutant complexes is drastically reduced if the basic residues are mutated. In addition to the basic residues in the α3 helix, Tyr 192, and Arg169 were found to be crucial for the stability of the VncR-*cpsp* complex (Supplementary Figure S7).

## Discussion

Comparative sequence analysis of VncR exhibited a great similarity to the PhoB of other bacteria (Throup et al., 2000), which has been known to regulate the expression of various bacterial virulence factors (McCluskey et al., 2004). Our results suggested that VncR plays a role in the induction of only type 2 and type 6B pneumococcal *cps* gene expression in the presence of serum and LF is the main factor behind this induction. We found that the CPS levels were significantly increased both in the type 3 strain and its *vncR* deletion mutant after a 2 h of serum treatment, although we could not detect the increase in *cps* genes mRNA levels of type 3 and its vncR deletion mutant after 2 h of serum exposure. The disparity of this VncR-regulated *cps* transcription may be owed to the fact that serotype 3 strains usually form a simpler polysaccharide structure using a distinct synthesis mechanism (Yother, 2011). Our study proposes that, when pneumococci get exposed to host serum during invasion, VncS senses LF, a serum component, resulting in the induction of VncRS. Induced VncR then binds to the promoters of *cps* genes to regulate their expression. In line with our *in vitro* data, we found that mice infected with D39Δ*vncR* and BG7322Δ*vncR* showed significantly higher survival rates than mice infected with their respective WTs, but the WU2Δ*vncR* showed no difference. This may be attributed to the comparatively lesser amounts of CPS of D39Δ*vncR* and BG7322Δ*vncR* in serum, which makes them less resistant to opsonophagocytosis, as CPS expression uplifts resistance to the complement C3 deposition and, consequently, to neutrophil-mediated phagocytosis (Kim et al., 1999; Hyams et al., 2010).

In contrast, EMSA results indicated that in presence of serum, the DBD of the VncR protein can bind to the *cpsp* of all these three serotypes, suggesting that bacterial transcription is dependent upon a coordinate regulation of various factors (Balleza et al., 2009), and that the DNA-binding protein primarily binds somewhere along the DNA non-specifically, followed by diffusion in search of its specific target (Li and Xie, 2011). In agreement with our previous report (Lee et al., 2018), this study proposes that when pneumococci get exposed to host serum during invasion, VncS senses the serum component LF, which results in the phosphorylation of VncS. Phosphorylated VncS then provides a phosphate group to VncR, and phosphorylated VncR binds to the *cpsp*, which permits the pneumococci to acclimatize to the blood environment. To best mimic *in vivo* conditions, a purified C-terminal DNA-binding domain of VncR was used in EMSA, so it can bind target DNA without being phosphorylated as the N-terminal domain removal makes it active (Makino et al., 1996). Our findings clearly have some limitations, as it cannot disclose the actual underlying mechanism of this serum-dependent interaction between VncRc with bacterial DNA, and further studies need to be carried out for revealing the underlying mechanism. Under these circumstances, we assume that the presence of serum albumin, which has an amazing ligand-binding capacity and is known to confer stability to many serum components (Sleep, 2015) might somehow facilitate the VncRc-*cpsp* interaction.

To speculate this differential expression of VncR-induced *cps* genes in type 2, 3, and 6 strains, we proposed a VncR-DNA model. Interestingly, type 2 and 6 *cpsp* contain a regular direct repeat, which is absent in type 3. However, all three serotypes share identical VncR protein structures. Thus, we propose that like type 2 and type 6, type 3 may also establish contact with *cpsp*, supporting our EMSA results, possibly owing to the identical DNA-binding α3, containing positively charged residues and the helix in serotype 3. However, due to the lack of VncR-specific repeats in the type 3 pneumococcal *cpsp* region, type 3 VncR had the least probability of regulating underlying *cps* transcription. To further validate our proposed model, the VncR-*cpsp* complex was simulated under physiologic conditions. The proposed tandem complex retained its stability throughout the simulation. Our *in silico* molecular simulation data provide evidence in favor of VncR-regulated strain-specific CPS synthesis in the presence of serum, which ultimately influences virulence.

Taken together, our results offer solid indication that pneumococci can regulate *cps* gene expression strain-specifically in order to familiarize with the varying host environment during invasion through VncR-*cpsp* interaction. Considering the importance of pneumococcal CPS in invasive diseases, it would be a practical approach to design small molecular inhibitors that target VncR-regulated encapsulation to combat pneumococcal infection.

## Data Availability Statement

All datasets generated for this study are included in the manuscript/Supplementary Files.

## Ethics Statement

All experimental procedures were reviewed, approved, and sanctioned by the Ethical Committee of Sungkyunkwan University, based on the Helsinki Declaration.

## Author Contributions

DR and PG conceived the experiments. PG, MS, SR, S-SP, and HI performed the experiments. PG, MS, SR, SC, KK, and DR analyzed the data. PG, MS, and DR wrote the manuscript. All authors read and approved the final manuscript.

## Conflict of Interest

The authors declare that the research was conducted in the absence of any commercial or financial relationships that could be construed as a potential conflict of interest.
