# Supplementary material for: Pneumococcal VncR Strain-Specifically Regulates Capsule Polysaccharide Synthesis
Source: Front Microbiol. 2019 Oct 1;10:2279. doi: 10.3389/fmicb.2019.02279 (PMC6781885; doi:10.3389/fmicb.2019.02279)
Supplement: Supplementary file 1 [file Table_1.DOCX]

**Supplementary Table S1. Primers used for *vncR* deletion mutant construction:**

| **Primer name** | **Sequence (5’-3’)** |
| --- | --- |
| vncR-LEFT-F | CCCTCTTGCTCAGTCTTTGG |
| vncR-LEFT-R | ATCAAACAAATTTTGGGCCCGG-TCATAGCCACAATCCGTCAA |
| vncR-RIGHT-F | ATT CTA TGA GTC GCT GCC GAC T-ATT GGA GCG AAA ATG AAA CG |
| vncR-RIGHT-R | GTT CAT CCA CAA TCC CCA AG |
| ermB-F | CCG GGC CCA AAA TTT GTT TGA T |
| ermB-R | AGT CGG CAG CGA CTC ATA GAA T |

**Supplementary Table S2. Primers used for qRT-PCR study:**

| **Primer name** | **Sequence (5’-3’)** |
| --- | --- |
| cps2A-F | CGTCAACCGAAGCACTG |
| cps2A-R | GATCCATCCGACCTGTCC |
| cps2B-F | TCGTTATGCCTTGATAGAAT |
| cps2B-R | ATTTACTTGCGTGTAACAGC |
| cps2C-F | TTTGCAGGCAGGATCTTATC |
| cps2C-R | GGCTTCCTCTGGCTGTTTAT |
| cps2D-F | AATTCAGGCTGGCTCTGTGT |
| cps2D-R | TAATAGCCGCATCAATCACG |
| cps2E-F | ATGGGGGCTCTGCTATTTTT |
| cps2E-R | TCCACCCTGCATGGTATTTT |
| cps2F-F | GCTTTGGGAAAATTGTTTGC |
| cps2F-R | AACATTCCCACCGTGTTGAC |
| cps2G-F | GACGCCGTATTCCAGAAAAG |
| **Primer name** | **Sequence (5’-3’)** |
| cps2G-R | CCCCATGCTTCAATTTCACT |
| cps2H-F | ATGGACTGGCTGATGGTTCT |
| cps2H-R | TTGACACCACCAAATCCTGA |
| cps2I-F | CAGGCAGCAATCAAACAGAG |
| cps2I-R | AAATCAACCTGCCCTTTTCC |
| cps2J-F | TTTTGTGGTGTAGCGGTGAA |
| cps2J-R | ATTCCACCAGCAAATCCTGA |
| cps2K-F | TGAACGCTTGGTAAGTGCTG |
| cps2K-R | ACCGCCTCAGCTTCAGTAAA |
| cps2P-F | TTAAACGCCTTCCAGTTCGT |
| cps2P-R | CAATCCACTCCTAGCCCAAC |
| cps2L-F | GGCTTTGCTTGGCTTGATAC |
| cps2L-R | CCAACTCCAGCACATCTTCA |
| cps2M-F | CGTGGCTGGTTTAAGGAAAA |
| cps2M-R | TCAACCCAAGAACCCAGAAC |
| cps2N-F | CCAAGCTCGCCTTACTCATC |
| cps2N-R | AGTTGGAAATCGTTGCCTTG |
| cps2O-F | GTTGATGCAGCAGAGGATGA |
| cps2O-R | CCCACTCTTGTCCAACTGGT |
| Cps3D-F | AAAAGAGCATGGCAGTTTGC |
| Cps3D-R | GCAATACCCTCCGTAACCAA |
| Cps3S-F | GCAACTAAGGTGGGCAGAAG |
| Cps3S-R | AATTTCCCACCATGAAGCTG |
| **Primer name** | **Sequence (5’-3’)** |
| wzg-F | TGTCAGCTCTGTGTCGCTCT |
| wzg-R | TCTCCCCTGCAATCAAACTC |
| wzh-F | TACTCCAGTCATTGCCCACA |
| wzh-R | TGGGTTTGAGGACATGTGAA |
| wzd-F | TTTCTTGGAGGAGCAGTCGT |
| wzd-R | GGTGTCGAGCAACTCAATCA |
| wze-F | AATTCAGGCTGGCTCTGTGT |
| wze-R | ACCTGCCTCCGTCACTAAAA |
| wchA-F | ATGGGGGCTCTGCTATTTTT |
| wchA-R | TCCACCCTGCATGGTATTTT |
| wciN-F | CAAAACAATTTGCGCAGAGA |
| wciN-R | CCAGCATTAAACATTGGCTTG |
| wciO-F | GCCAGATATCGGGAGCAATA |
| wciO-R | AGCCAGCCATTGCATCTACT |
| wciP-F | GGGGATTGAATTACCGAACA |
| wciP-R | CCCCCAGATATAGGCATGAA |
| wzy-F | GTTTGGGGAACAGAACTCCA |
| wzy-R | ATGCGAAATTCCCTCCACTT |
| wzx-F | CGGGATGCGTTGATTTTTAT |
| wzx-R | TTCCATCCTTAGCGCCTTTA |
| rmlA-F | GGTCCTGGTCTGAGCACAAT |
| rmlA-R | CGCACCTTTCTCTTTCTTGG |
| rmlC-F | CGCAAGGTTGAAGCTATTCC |
| **Primer name** | **Sequence (5’-3’)** |
| rmlC-R | CCAGCCACGATTATCTCCAT |
| rmlB-F | GTTGGTGACATTGCTGATGC |
| rmlB-R | CTGGTCCTTCACCATGACCT |
| rmlD-F | GTTGATGCAGCAGAGGATGA |
| rmlD-R | CCCACTCTTGTCCAACTGGT |

**Supplementary Table S3. Primers used to show the VncR-*cpsp* interaction:**

| **Primer name** | **Sequence (5’-3’)** |
| --- | --- |
| cpsp-F | TACACATCTGCTTCTAAAATATTGT |
| cpsp-R | TTAAAACGTCTACTCATGATTAACA |
| cps3p-F | TATCTTTTCAAAGCTGATACTAAGG |
| cps3p-R | TTTACTACAGTATTTCTCTGCA |

**
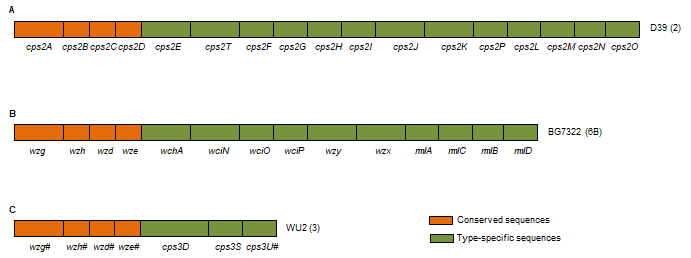
Supplementary Figure S1: Schematic representation of *cps* genes cluster of *S. pneumoniae* strains D39 (type 2; A), BG7322 (type 6B; B), and WU2 (type 3; C).** The *cps* genes have been named according to BPGN system. # signifies either the genes are mutated or their transcription is not necessary for CPS biosynthesis.

**
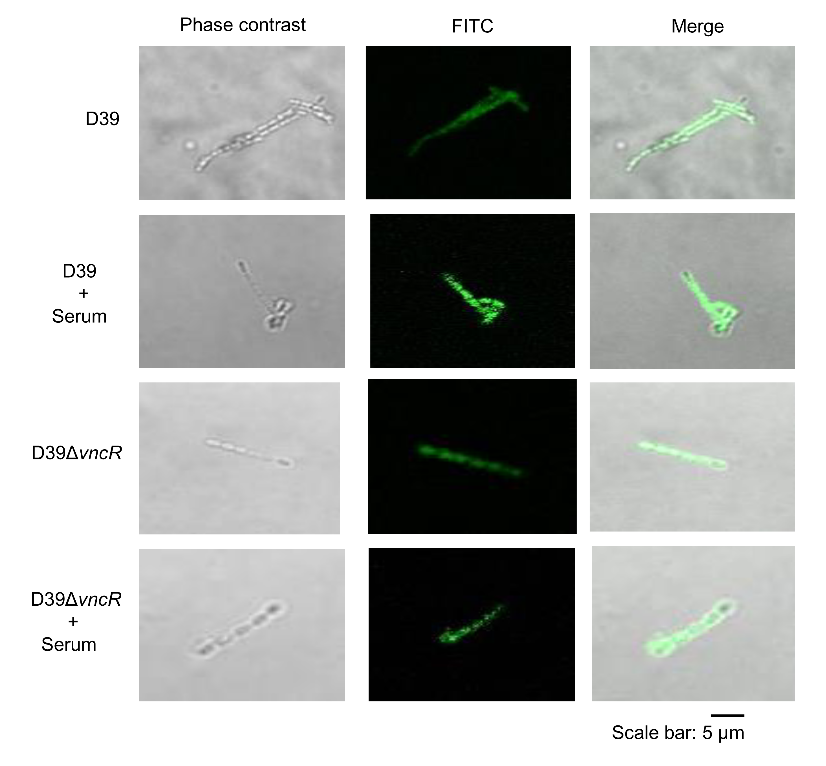
**

**Supplementary Figure S2: Deletion of *vncR* inhibits type 2 pneumococcal CPS expression in the presence of serum.** FITC-labelled CPS of *S. pneumoniae* WT D39 and its isogenic *vncR* mutant was visualized by confocal microscopy, after 20 min serum supplementation (Scale bar: 5 µm). The data is representative of two independent experiments.

**
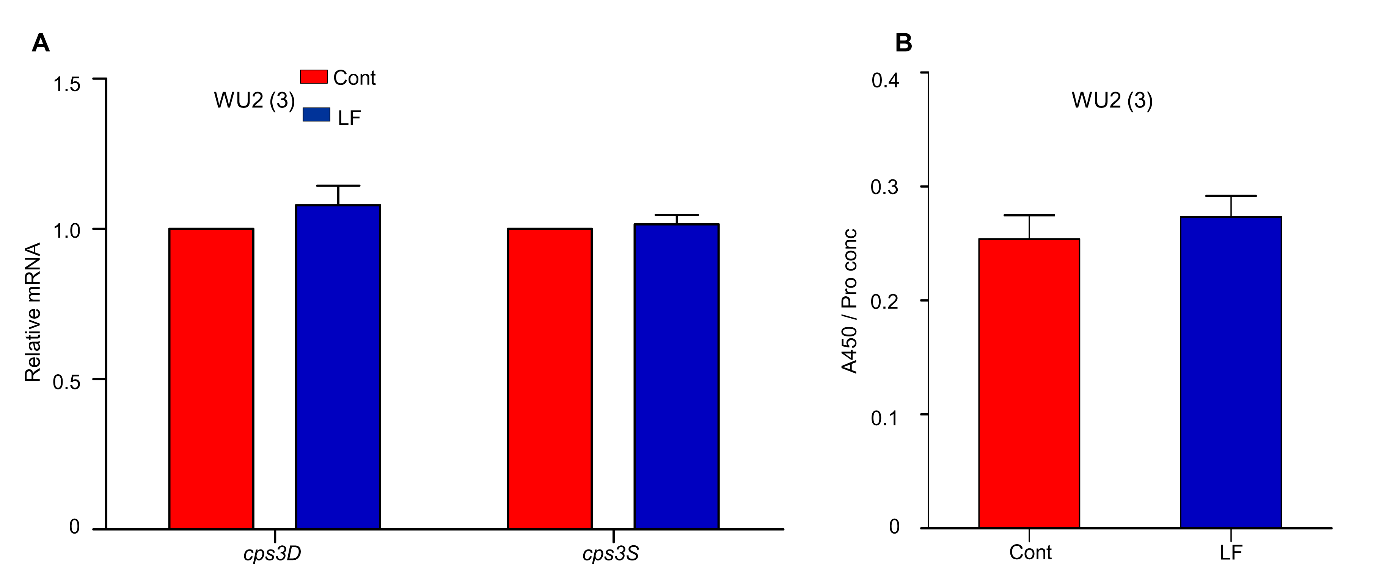
Supplementary Figure S3: Lactoferrin (LF) does not have any effect on the CPS synthesis of type 3 pneumococci:** The WU2 (type 3) strain was incubated for 2 h with LF. The mRNA expression levels of the *cps* genes were analyzed by qRT-PCR (**A**). Total CPS quantities in whole cell lysates were determined using ELISA after cross-reacting with the type-specific CPS antiserum (**B**). Data are expressed as mean ± standard error of mean (SEM) of 3 experiments in quadruplicates.

**
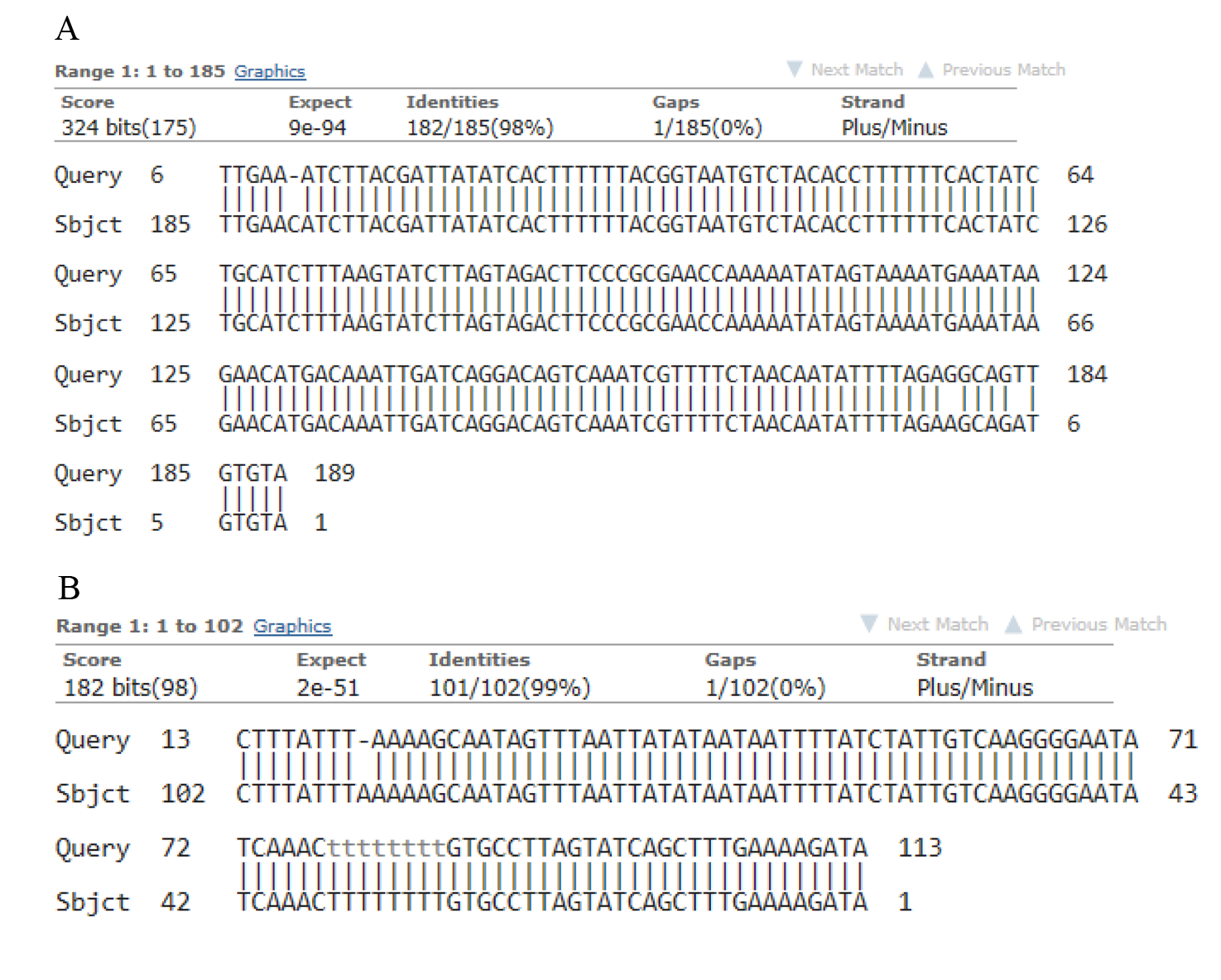
Supplementary Figure S4: Sequencing analysis of co-immunoprecipitated complex.** Sequences aligned to the reference sequences that confirm the presence of specific D39 (**A**) and WU2 (**B**) *cps* promoters in co-IP complex.

**
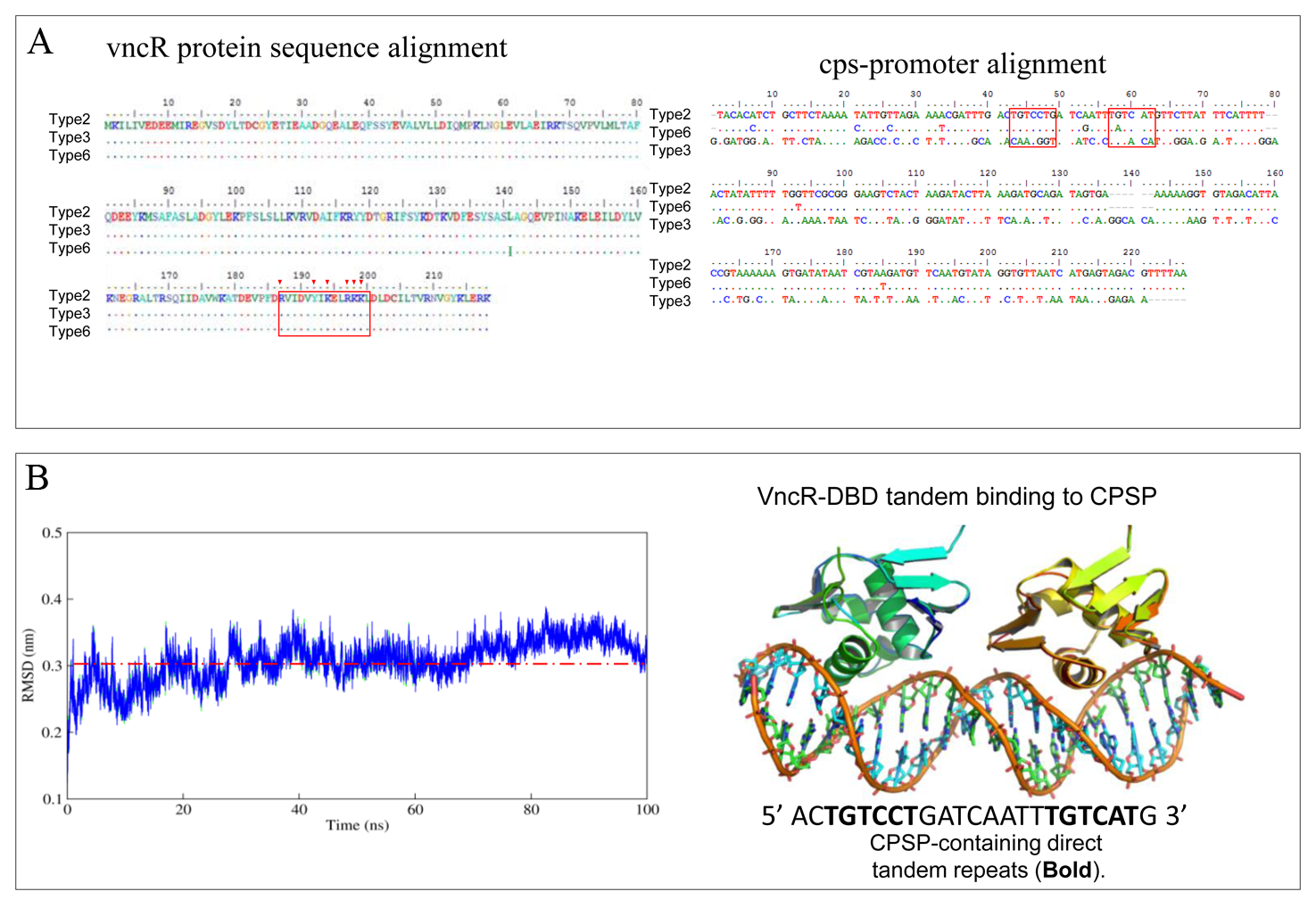
**

**Supplementary Figure S5: Molecular dynamics simulation (MDS) of the VncR-DBD-DNA complex and *cps*-promoter analysis.** Alignment of the amino acid sequences of the VncR type 2, type 3, and type 6. This alignment suggests that all these types share identical sequences, specifically helix α3 which contains positively charged DNA-interacting residues (Red box and red arrow heads above). The nucleotide sequences of the *cps*-promoter regions of type 2 and type3, and types 6 are aligned to indicate the direct tandem repeats. The boxes indicate the presence of direct repeats in type 2 and 6, but not in type3 (**A**). The RMSD plot indicates a stable oscillation of the backbone atoms around 3Å as compared to the initial conformation of the complex. Cartoon representation indicates a superimposed model of the before MDS and a stable VncR-DNA complex taken from MDS (**B**).

**
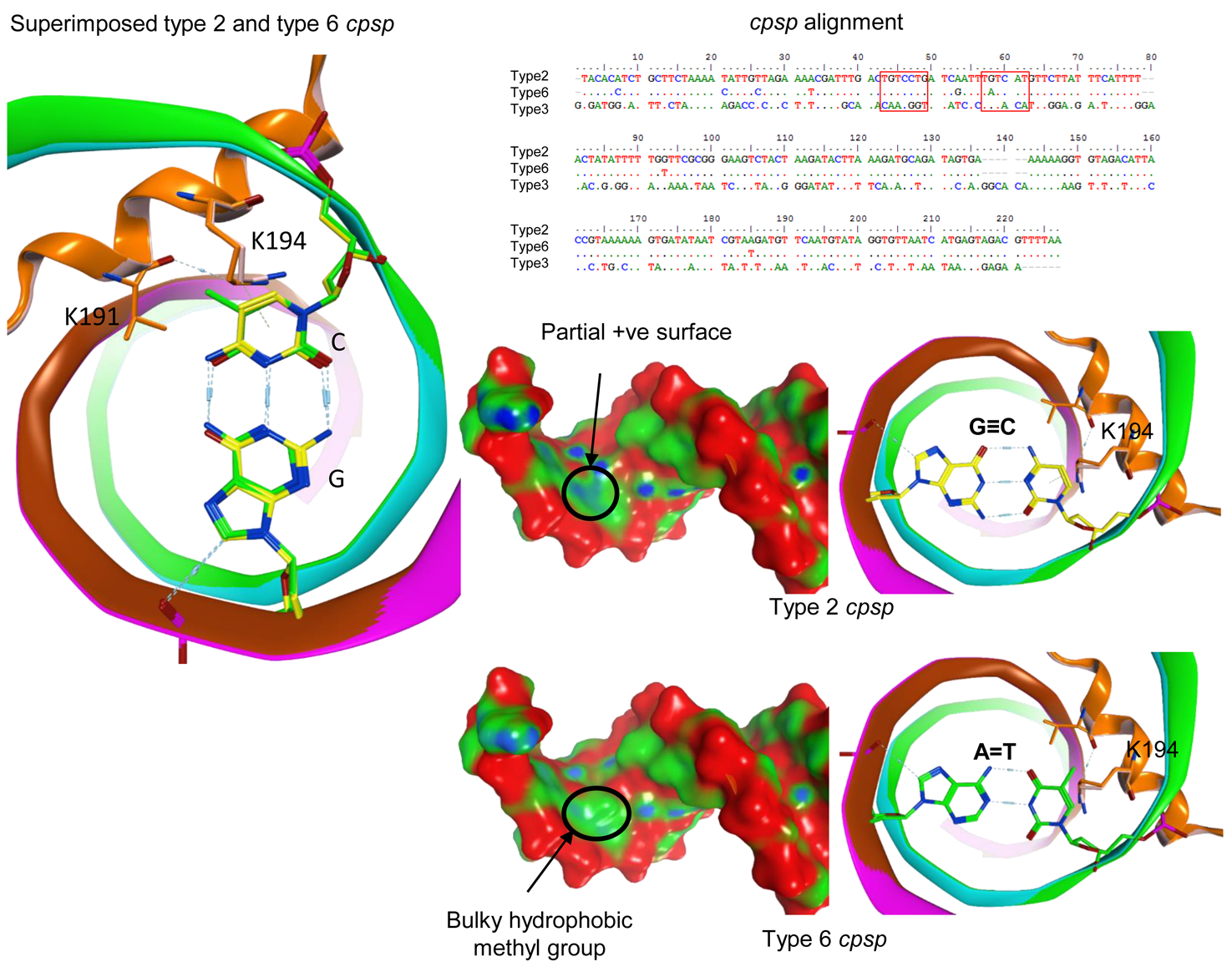
**

**Supplementary Figure S6: Type 2 and type 6 *S. pneumoniae* contain direct tandem repeats, TGTCC/AT, important for the VncR-dependent transcription regulation of CPS.** In type 6 *cpsp,* conserved guanosine is mutated into adenosine (green colored sticks). This mutation creates an additional hydrophobic patch (encircled green patch shown in the electrostatic surface map of *cpsp*) in the major grove of *cpsp* that is essential for α3 helix binding. This mutation disrupts the cationic-π interaction that is important for VncR-*cpsp* interaction and stability.

**
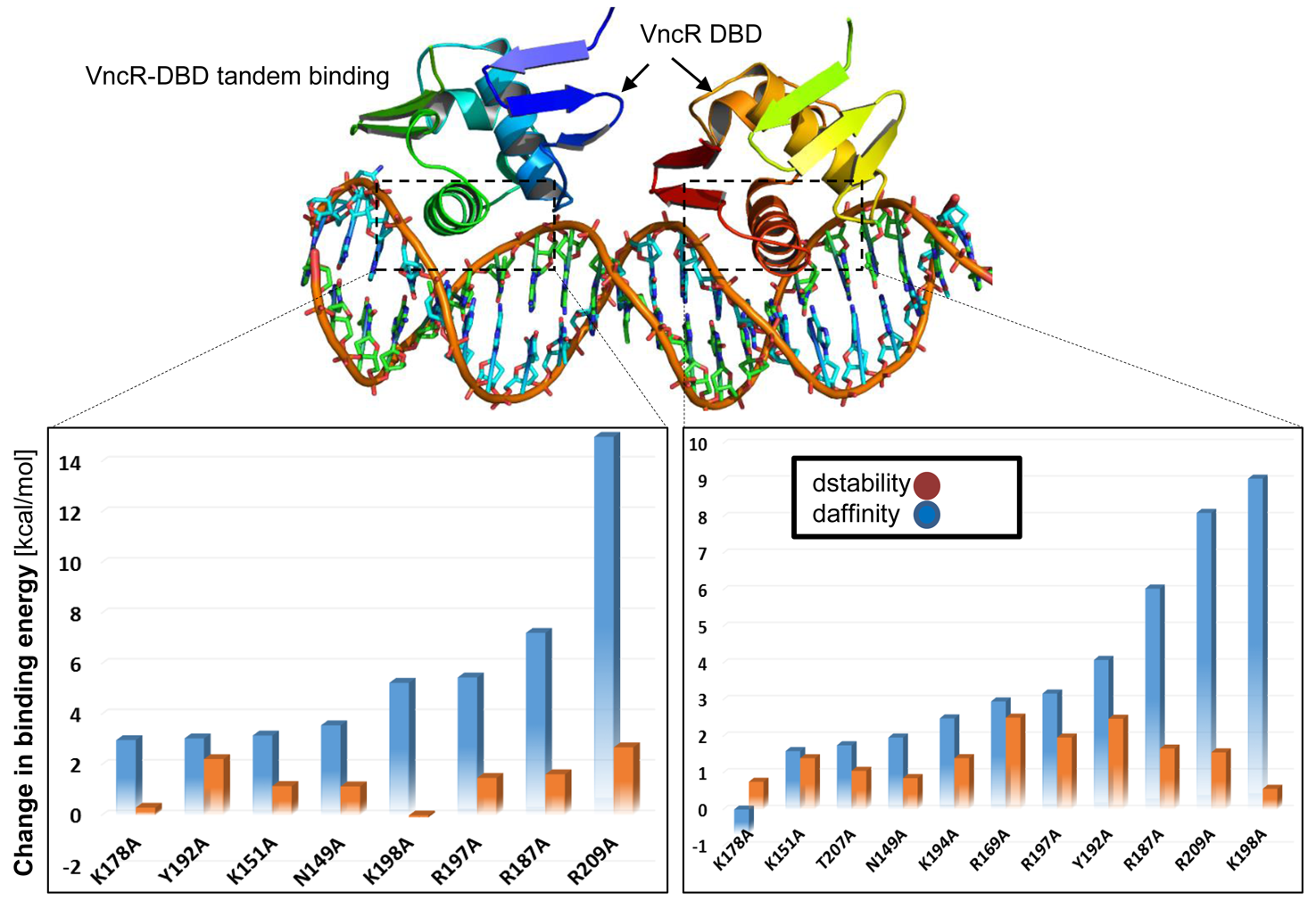
**

**Supplementary Figure S7: Computational alanine scanning of the VncR-DNA interfacial residues.** The changes in the DNA-binding affinity and stability of the complex (daffinity and dstability; relative changes in the affinity of VncR towards DNA and the overall stability of the complex, in terms of energies) were measured as differences in binding energy of the WT hot spot residues in the VncR-DBD-DNA complex to the alanine mutant. The potential hot spot residues are indicated as positive high binding free energy difference.
